# Quantitative Perfusion Analysis of First-Pass Contrast Enhancement Kinetics: Application to MRI of Myocardial Perfusion in Coronary Artery Disease

**DOI:** 10.1371/journal.pone.0162067

**Published:** 2016-09-01

**Authors:** Sohae Chung, Binita Shah, Pippa Storey, Sohah Iqbal, James Slater, Leon Axel

**Affiliations:** 1 Center for Advanced Imaging Innovation and Research (CAI2R), New York University School of Medicine, New York, NY, 10016, United States of America; 2 Department of Radiology, Bernard and Irene Schwartz Center for Biomedical Imaging, New York University School of Medicine, New York, NY, 10016, United States of America; 3 Department of Medicine, Division of Cardiology, New York University School of Medicine, New York, NY, 10016, United States of America; Jilin University, CHINA

## Abstract

**Purpose:**

Perfusion analysis from first-pass contrast enhancement kinetics requires modeling tissue contrast exchange. This study presents a new approach for numerical implementation of the tissue homogeneity model, incorporating flexible distance steps along the capillary (NTH_*f*_).

**Methods:**

The proposed NTH_*f*_ model considers contrast exchange in fluid packets flowing along the capillary, incorporating flexible distance steps, thus allowing more efficient and stable calculations of the transit of tracer through the tissue. We prospectively studied 8 patients (62 ± 13 years old) with suspected CAD, who underwent first-pass perfusion CMR imaging at rest and stress prior to angiography. Myocardial blood flow (MBF) and myocardial perfusion reserve index (MPRI) were estimated using both the NTH_*f*_ and the conventional adiabatic approximation of the TH models. Coronary artery lesions detected at angiography were clinically assigned to one of three categories of stenosis severity (‘insignificant’, ‘mild to moderate’ and ‘severe’) and related to corresponding myocardial territories.

**Results:**

The mean MBF (ml/g/min) at rest/stress and MPRI were 0.80 ± 0.33/1.25 ± 0.45 and 1.68 ± 0.54 in the insignificant regions, 0.74 ± 0.21/1.09 ± 0.28 and 1.54 ± 0.46 in the mild to moderate regions, and 0.79 ± 0.28/0.63 ± 0.34 and 0.85 ± 0.48 in the severe regions, respectively. The correlation coefficients of MBFs at rest/stress and MPRI between the NTH_*f*_ and AATH models were r = 0.97/0.93 and r = 0.91, respectively.

**Conclusions:**

The proposed NTH_*f*_ model allows efficient quantitative analysis of the transit of tracer through tissue, particularly at higher flow. Results of initial application to MRI of myocardial perfusion in CAD are encouraging.

## Background

Given the high morbidity and mortality of coronary artery disease (CAD), which is largely related to associated disorders of the blood flow to the heart, it is very important to be able to assess the perfusion of the myocardium for the diagnosis and risk stratification of patients with suspected CAD. Cardiovascular magnetic resonance (CMR) has been used to perform myocardial first-pass perfusion imaging over the past decade.[1] The kinetics of the tissue enhancement after the injection of a contrast agent, both at rest and under stress, are key factors for diagnosis. The observed time course of the contrast enhancement of the heart can be analyzed using the principles of tracer dynamics, considering the contrast agent as a tracer. The tracer transit reflects both the plasma transit through the tissue and any exchange of tracer between the capillaries and the extravascular space [2–5]; this exchange depends on the permeability of the barrier between the vascular and extravascular spaces and on the sizes of those spaces, as well as on the flow rate through the vascular space. In general, there is a partial extraction (usually by passive diffusion) of the tracer from the vascular space into the extravascular space during the initial passage of the bolus; this then diffuses back into the vascular space as the intravascular concentration drops.[6–8] The tissue homogeneity (TH) model has been used to estimate the perfusion of the myocardium by considering the extravascular space as a single effective tracer concentration at a given time [9], with which the intravascular spaces can exchange. Since the conventional TH model has no general analytic solution, an adiabatic approximation of the TH (AATH) model can be used, considering the concentrations to be effectively constant over finite time steps.[10, 11] However, when using the conventional AATH approach, with a fixed discretization of steps in space and time, estimation of parameters such as the capillary mean transit time can be problematic.[12]

In this study, we present a new numerical approach to the use of the TH model, incorporating flexible distance steps along the capillary (NTH_*f*_) and also including the contrast agent bolus arrival time between the site of observation of the arterial input function (AIF) and the tissue bed, to achieve more accurate and precise estimation of perfusion-related parameters. This approach allows for robust and accurate quantification of perfusion, by maintaining the detailed balance between the advective exchange in the capillary compartments, due to flow carrying the tracer into and out of each compartment at different concentrations, and the diffusive exchange between the vascular and extravascular compartments. Using a fast saturation-recovery multi-slice calibrated CMR method [13], AIF was measured precisely within the aortic root at a short delay time (TD) after the saturation pulse, in order to avoid signal clipping with high contrast agent concentrations in the blood, while myocardial wall images were acquired at longer TDs, in order to achieve higher SNR and good sensitivity to the wall contrast enhancement. Myocardial perfusion parameters were calculated with the NTH_*f*_ model from rest and stress first-pass contrast-enhanced CMR in patients with suspected CAD; the results were compared both with the corresponding perfusion parameters calculated with AATH and also with the clinical results from diagnostic invasive coronary angiography performed on the same day as the MRI examination.

## Methods

### Numerical Approach to the TH model with Flexible Distance Steps Along the Capillary (NTH_*f*_)

**Fig 1:**
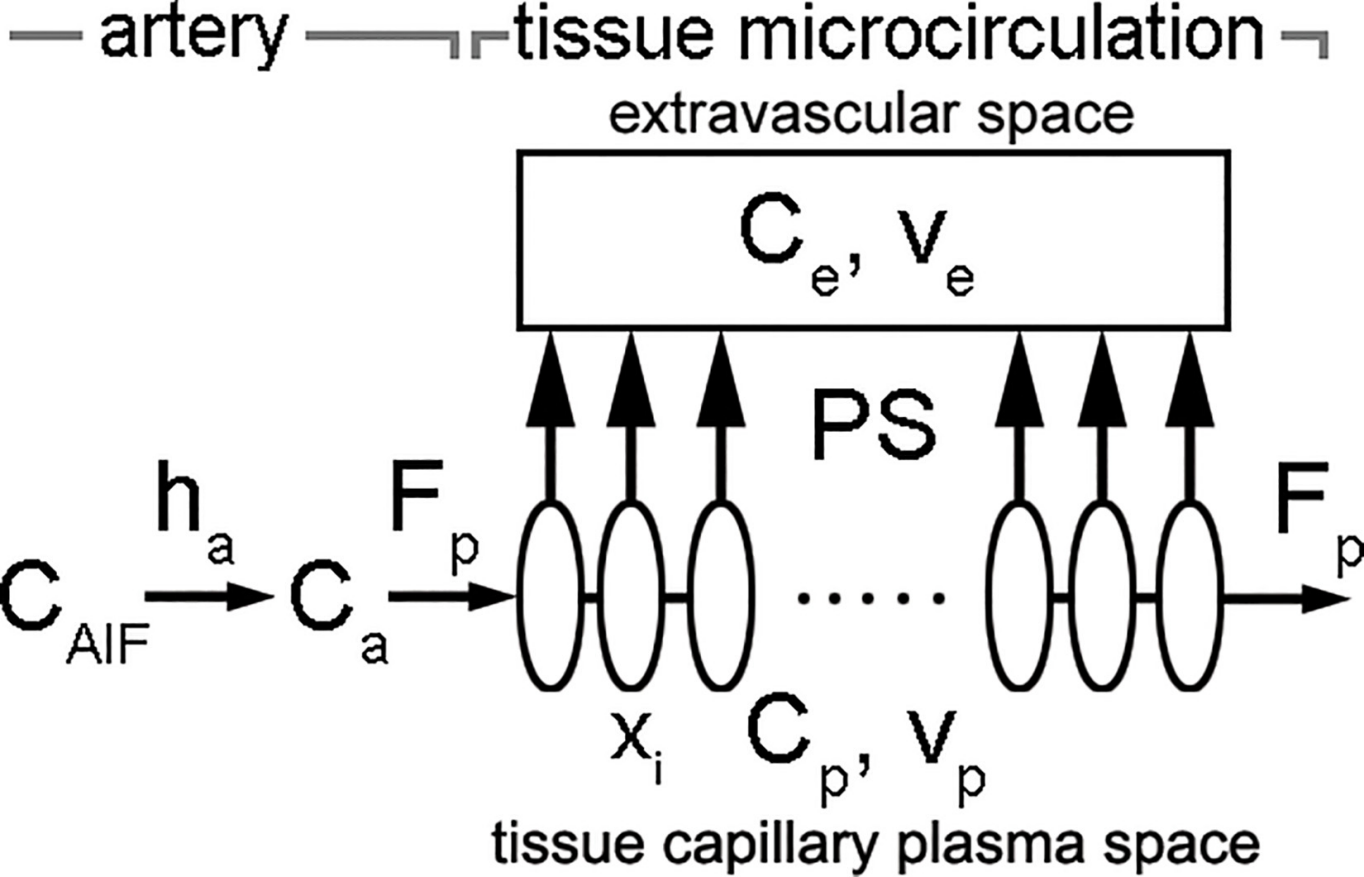
Schematics of the TH-based exchange model. C_AIF_, blood concentration of AIF; *h*_*a*_, arterial transfer function; *C*_*a*_, concentration of the arterial plasma input to the tissue; *F*_*p*_, plasma flow per unit tissue volume; *C*_*p*_, concentration in the tissue capillary plasma space; *C*_*e*_, concentration in the extravascular space; *v*_*p*_, plasma volume in the tissue; v_e_, extravascular interstitial volume; PS, product of the permeability and the surface area between the compartments; *x*_*i*_, a position along the capillary.

Fig 1 presents a schematic diagram of the TH-based exchange model. The abbreviations used in this section are summarized in Table 1. From the observed blood tracer concentration of an upstream AIF, *C*_*AIF*_(*t*), we estimate an arterial plasma input delivered to the tissue, *C*_*a*_(*t*), through a convolution by an arterial transfer function, *h*_*a*_(*t*,*τ*), to account for delay and prolongation of the tracer bolus between the point of observation and the tissue (e.g., characterized by a bolus arrival time, *τ*), including correction for the large vessel hematocrit (Hct) for plasma concentration:

$$C_{a}(t)=C_{AIF}(t)*\frac{h_{a}(t,\tau )}{1-Hct}.$$ (1)

Since there is only limited experimental data to draw on, *h*_*a*_(*t*,*τ*) is estimated by using a model of lagged normal density curves, resulting from the convolution of a Gaussian with a decaying exponential, scaled by a delay parameter, *τ*.[14] Note that we primarily consider plasma transport here, as the tracer is assumed to be dissolved only in the plasma space of the blood.

**Table 1:** Definitions of perfusion parameters.

Perfusion parameter	Definition	Unit
F_p_	Plasma flow per unit tissue volume	ml/cc/sec
v_e_	Extracellular extravascular volume fraction of tissue	
v_p_	Plasma volume fraction of tissue	
0030	Permeability surface area product	ml/g/min
Τ	Bolus arrival time	s
MBF	Myocardial blood flow per unit mass of tissue	ml/g/min

Given a position along the capillary (as shown in Fig 1), *x* = *x*_*i*_, and time, *t* = *t*_*j*_, we define *J*(*x*_*i*_,*t*_*j*_) as the passive diffusive flux of tracer between the tissue capillary plasma space and the extravascular space, i.e., *J*(*x*_*i*_,*t*_*j*_) = *PS* ∙ (*C*_*p*_(*x*_*i*_,*t*_*j*_) − *C*_*e*_(*t*_*j*_)), where PS is the product of the permeability and the effective surface area of the membrane between the compartments, *C*_*p*_(*x*_*i*_,*t*_*j*_) is the concentration in the tissue capillary plasma space, and *C*_*e*_(*t*_*j*_) is the concentration in the extravascular space. Following the TH model, we assume that *C*_*e*_(*t*_*j*_) does not depend on position. A conventional TH model considers the exchange of tracer between the flowing plasma and the extravascular space from a spatially fixed, “Eulerian” frame of reference, balancing the tracer fluxes across discrete segments of the capillary from advection along the capillary and diffusion across the wall of the capillary; this can lead to numerical problems at high flow rates. Instead, we consider the exchange from a “Lagrangian” frame of reference traveling with the plasma. That is, we estimate the exchange over a flexible discrete fractional distance traveled along the capillary by a plasma element and assume: 1) the extravascular concentration is uniform and effectively constant during a time step, Δ*t*, and 2) the capillary plasma concentration within the plasma element is effectively constant during Δ*t* at a given location, while the mean transit time (MTT) (the average time taken by the tracer to travel through the tissue) is estimated iteratively. Then, *C*_*p*_(*x*_*i*_,*t*_*j*_) and *C*_*e*_(*t*_*j*_) are calculated numerically from *C*_*a*_(*t*) and a given set of values of the model parameters, given the boundary condition *C*_*p*_(0,*t*) = *C*_*a*_(*t*):

$$C_{p}(x_{i},t_{j+1})=C_{p}(x_{i}-\delta_{x},t_{j})-\frac{1}{v_{p}}J(x_{i},t_{j})\delta_{x}\Delta t,$$ (2)

$$C_{e}(t_{j+1})=C_{e}(t_{j})+\frac{1}{v_{e}}\frac{\sum_{i}J(x_{i},t_{j})}{N}\delta_{x}\Delta t,$$ (3)

where *v*_*p*_ is the fractional plasma volume in the tissue, *v*_*e*_ is the fractional extravascular interstitial volume, *δ*_*x*_ = Δ*t*/*MTT* is the fractional distance traveled along the capillary by a plasma element during Δ*t* and *MTT* = *v*_*p*_/*F*_*p*_, *F*_*p*_ is the plasma flow per unit tissue volume, and N is the number of discretization intervals of x. We approximate the extravascular concentration as being effectively constant during Δ*t*. The net concentration of tracer in the capillary-tissue unit, *C*_*t*_(*t*), is calculated by summing over the capillary elements, together with the extravascular space:

$$C_{t}(t)=v_{p}\frac{\sum_{i}C_{p}(x_{i},t)}{N}+v_{e}C_{e}(t).$$ (4)

A nonlinear least-squares solver is used to find optimal parameter values (*F*_*p*_, *v*_*p*_, *v*_*e*_, PS and *τ*) to minimize the mean squared difference between the predicted *C*_*t*_(*t*) and the observed tissue concentrations, given the observed AIF. Maximum number of iterations is set to 400 and the termination tolerance is set to 10^−6^. In order to convert the plasma concentrations and flow to equivalent blood concentrations and blood flow, the value of Hct can be measured by sampling blood from a large vessel. However, the capillary hematocrit (Hct_c_) would generally be lower than the large vessel value, due to the fact that red cells transit more rapidly than plasma through the microcirculation [15]. Unfortunately, Hct_c_ is not readily measured, so we instead rely on estimates derived from the literature [16] to scale the large vessel hematocrit values to estimated equivalent capillary values (*Hct*_*c*_ = 0.7 × *Hct*). Thus, for application of the analysis to myocardial perfusion calculation, the myocardial blood flow through the tissue per unit mass of tissue (MBF) (ml/g/min), was calculated by $MBF=\frac{F_{p}}{(1-{Hct}_{c})∙\rho }∙60$, where *ρ* is the assumed tissue density of 1.05 (g/cc) (for *F*_*p*_ in units of ml/cc/sec). Myocardial perfusion reserve index (MPRI) was calculated by dividing results of myocardial perfusion at stress by results at rest, with an associated independence from many common constant factors.

### Study Population

This study was approved by our Institutional Review Board at the NYU School of Medicine, and all subjects provided written informed consent before the procedure. We prospectively studied 8 patients (6 male; age, 62 ± 13 years old; body mass index, 29.4 ± 4.3 kg/m^2^) with suspected coronary artery disease (CAD), who underwent first-pass perfusion rest and stress CMR imaging (described below), followed by a clinical diagnostic invasive coronary angiography examination on the same day. Exclusion criteria included unstable angina, irregular heart rate, valvular heart disease, and contraindications for MRI examinations (e.g., pacemakers or ferromagnetic vascular clips), contrast agent injection (e.g., contrast allergies), or pharmacologic stress agents (e.g., significant asthma when undergoing regadenoson stress).

### CMR Imaging

All subjects were instructed to refrain from foods and beverages containing caffeine for 12 hours prior to the procedure. Imaging was performed on a 3T whole-body MR scanner (Tim Trio; Siemens Medical Solutions, Erlangen, Germany), using a standard phased-array coil that was applied to the anterior chest of the patient in the supine position. Blood pressure, heart rate, and electrocardiogram were monitored during the CMR examination. Rapidly repeated T_1_-weighted (T_1_w) CMR images were acquired during a contrast agent bolus passage at four slice locations in each heart beat: aortic root, for measuring the AIF, and short-axis slices at three levels of the left ventricle, for measuring the wall response. A first perfusion scan was performed at rest, followed by a second scan, after a delay of 10 minutes to allow for the initial clearance of the first contrast agent injection, during maximal vasodilatation. Maximal vasodilatation was obtained about 90 seconds after infusion of 0.4 mg regadenoson (Lexiscan 0.4 mg IV bolus, Astellas Pharma, USA) over 10 seconds. Each set of perfusion images was acquired during breath-holding for 10 heartbeats before a contrast injection, to acquire baseline T1w images, and for 40 heartbeats during the first-pass transit of a bolus of 0.05 mmol gadolinium-diethylenetriamine pentaacetic acid (Gd-DTPA)/kg body weight (0.5M Berlex Magnevist, Schering AG, Germany), injected at an injection rate of 5 mL/s, followed by a 20 mL bolus of normal saline flush. If breath-holding could not be sustained for the full duration of the data acquisition, subjects were allowed shallow breathing during the remainder of the imaging (respiratory motion was manually compensated for during image analysis; see below).

A cardiac-triggered saturation-recovery (SR) ultrafast gradient echo (i.e., TurboFLASH) pulse sequence was used to sequentially acquire T_1_w images at multiple slices with sequential TDs after a single non-selective robust saturation pulse.[13, 17, 18] Imaging parameters included: FOV = 340–400 mm × 289–340 mm, slice thickness = 8 mm, in-plane resolution = 1.06–1.25 mm × 1.06–1.25 mm, TE/TR = 1.2/2.4 ms, flip angle = 10°, temporal resolution = 114–189 ms, generalized autocalibrating partially parallel acquisitions (GRAPPA) [19] with an effective acceleration factor ~ 1.65, centric k-space trajectory, and receiver bandwidth = 1008 Hz/pix. In order to correct for the spatially varying receive coil sensitivities and the unknown equilibrium magnetization, a proton density-weighted (PDw) image (flip angle = 5°) was acquired without the saturation pulse in the first heartbeat, which was used to normalize the subsequent images.[20] The resulting data were analyzed as previously described [13] to calculate the corresponding time course of contrast agent concentration in the blood and the myocardium, assuming rapid relaxation exchange between tissue water and the contrast agent and a known relaxivity of the contrast agent.

### Data and Statistical Analyses

**Fig 2:**
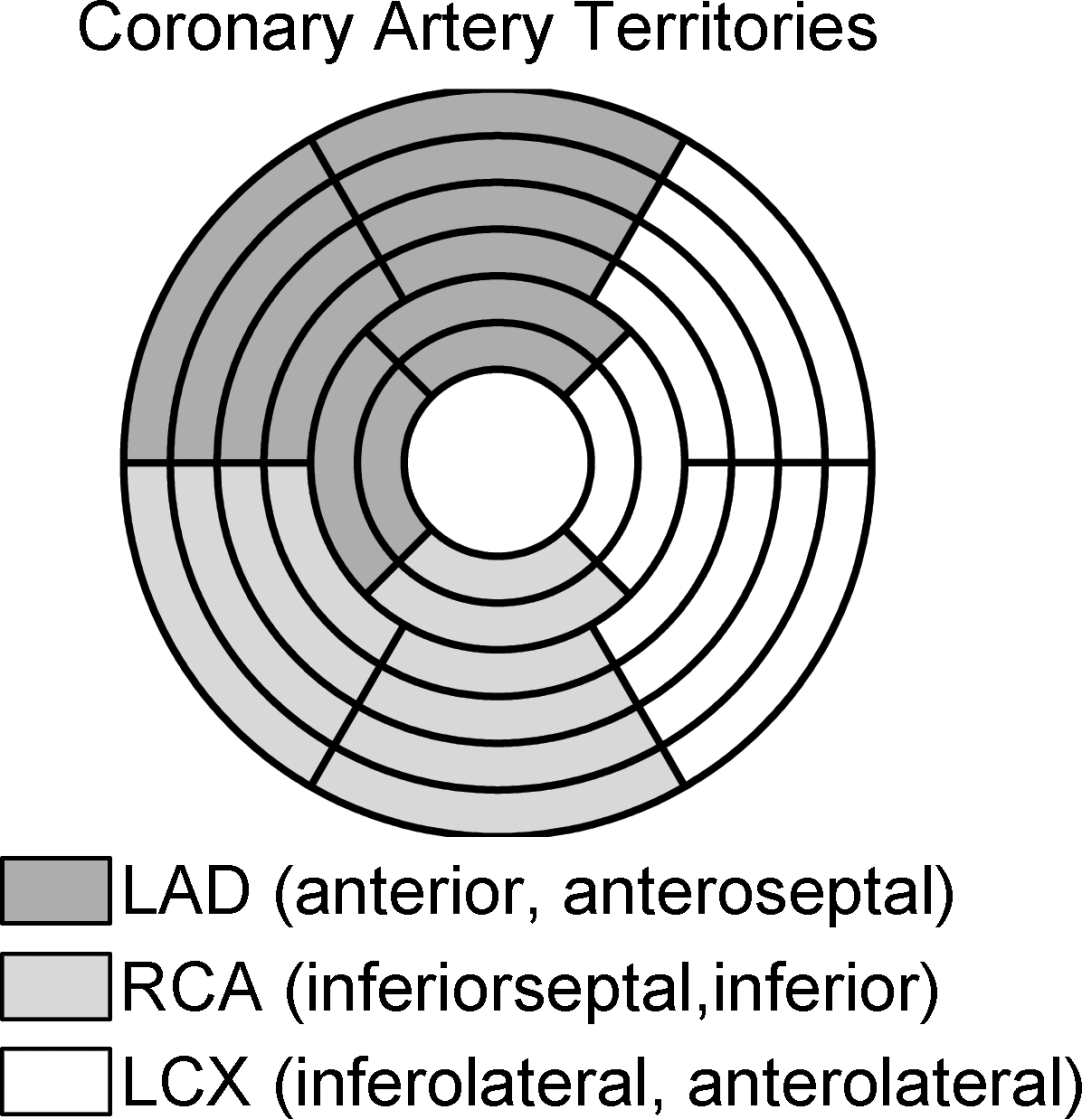
Display of the 16 cardiac short-axis segments divided into epicardial and endocardial regions (total 32 segments). Colors indicate the three coronary artery territories of the left anterior descending (LAD), right coronary artery (RCA), and the left circumflex coronary artery (LCX).

Data analysis was performed using custom software developed in MATLAB R2015a (The Mathworks, Inc., Natick, MA) on a 3.47 GHz Intel Core i7 personal computer with 24 Gb of RAM. As shown in Fig 2, a 16-segment model of the left ventricle (LV) based on the standard “17-segment” model (without the apical “cap”) was used, dividing the LV into 6 basal, 6 mid, and 4 apical segments.[21] These segments were also each further divided into epicardial (epi) and endocardial (endo) regions (total 32 segments). Tracking of the segments to compensate for respiratory motion was performed manually; the respiratory motion was assumed to be primarily in-plane. Individual myocardial segments were assigned to the territories of the three major coronary arteries (LAD, left anterior descending; RCA, right coronary artery; LCX, left circumflex) as per standard recommendations (Fig 2).[21] The severity of coronary artery stenosis was determined from the invasive coronary angiography examination by a cardiologist after the procedure and was expressed as a percent reduction in vessel diameter. The lesions were assigned to one of three categories of severity: 1) < 40% narrowing (insignificant), 2) 40% to 80% (mild to moderate), and 3) ≥ 80% (severe). Data are presented as mean ± standard deviation. Student’s t-test was used to compare the results. P values less than 0.05 were considered significant.

### Comparison of NTH_*f*_ and AATH Perfusion Estimates

For comparison, we used a conventional AATH model to estimate the following perfusion parameters: tissue blood flow, F, transit time through capillary, T_c_, capillary permeability-surface area product, PS, and rate constant for the clearance of the tracer, k_adb_.[11] *C*_*a*_(*t*) calculated from the results of the NTH_*f*_ analysis was used as an input function. The Spearman's correlation coefficient was used to evaluate the correlations between the MBFs found from the NTH_*f*_ and AATH models. The residual sum-of-squares (RSS) was calculated for each segment, as a measure of the goodness-of-fit, by using the following equation: *RSS* = ∑_*time*_(*observed data* − *estimated fit*)^2^.

### Noise Stability Estimation

For noise stability estimation of the NTH_*f*_, myocardial perfusion signals were simulated with a low MBF (= 0.8102 ml/g/min) and a high MBF (= 2.2006 ml/g/min). White Gaussian noise (WGN) sets were generated with a range of the signal-to-noise ratio (SNR) = 1 to 60 (dB) (2 dB steps), using a function (awgn) in MATLAB R2015a, and were added to the simulated [Gd-DTPA] signals. The simulated noisy signals were then used to calculate the estimated MBF. For each SNR value, a computation was repeated 30 times. Percent error (%) was calculated by |estimatedMBF-trueMBF|/trueMBF×100.

## Results

### NTH_*f*_

The mean MBF of all patient regions at rest supplied by coronary arteries with insignificant stenosis was 0.80 ± 0.33 ml/g/min, which increased to 1.25 ± 0.45 ml/g/min at vasodilator stress, resulting in an average myocardial perfusion reserve index (MPRI) of 1.68 ± 0.54. In regions supplied by vessels with mild to moderate stenosis, the mean MBF at rest (0.74 ± 0.21 ml/g/min) was similar to that in the insignificant stenosis regions, but was lower during vasodilator stress (1.09 ± 0.28 ml/g/min; p < 0.05), resulting in a slightly lower average MPRI of 1.54 ± 0.46 (p < 0.05). In regions supplied by vessels with severe stenosis, the mean MBF at rest (0.79 ± 0.28 ml/g/min) was again similar to that of the regions supplied by vessels with insignificant or the mild to moderate stenosis, but it was even lower during vasodilator stress (0.63 ± 0.34 ml/g/min; p < 0.05). The resulting MPRI (0.85 ± 0.48) was significantly lower than in regions supplied by vessels with mild to moderate stenosis (p < 0.001). The results of the NTH_*f*_ and AATH models are summarized in Table 2.

**Table 2:** NTH_*f*_ and AATH results: MBF and MPRI corresponding to percent diameter stenosis of coronary lesion.

		Percent Diameter Stenosis of Coronary Lesion		
		Insignificant (< 40%)	Mild to Moderate (40% to 80%)	Severe (> 80%)
MBF at rest	NTH_*f*_	0.80 ± 0.33	0.74 ± 0.21	0.79 ± 0.28
	AATH	0.79 ± 0.26	0.75 ± 0.15	0.79 ± 0.20
MBF at stress	NTH_*f*_	1.25 ± 0.45	1.09 ± 0.28	0.63 ± 0.34
	AATH	1.10 ± 0.30	0.99 ± 0.20	0.65 ± 0.31
MPRI	NTH_*f*_	1.68 ± 0.54	1.54 ± 0.46	0.85 ± 0.48
	AATH	1.45 ± 0.32	1.36 ± 0.29	0.83 ± 0.36

**Fig 3:**
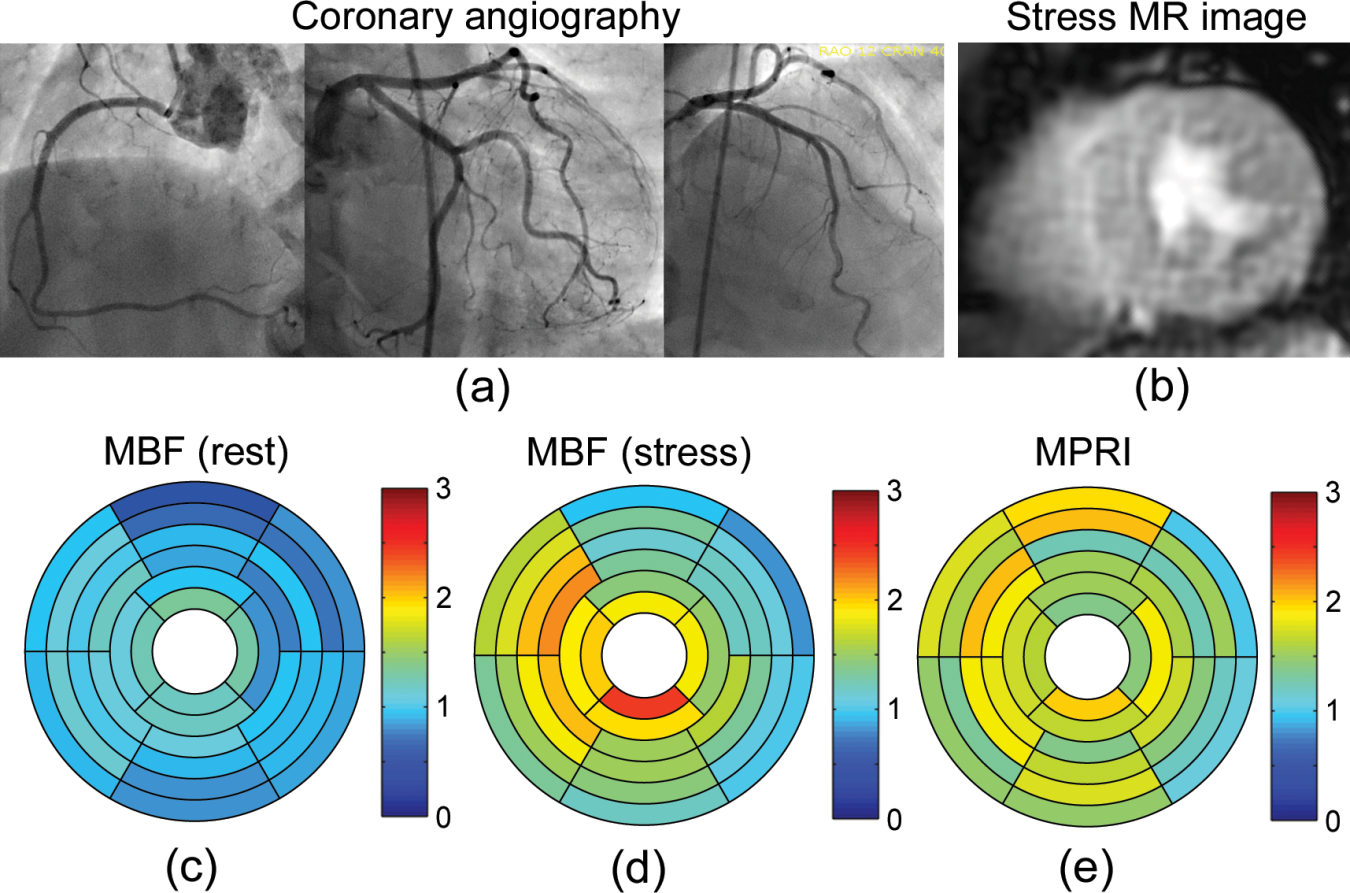
(a) Selective coronary angiography in a representative patient (83-year-old female) with angiographically normal coronary arteries. (b) First-pass perfusion mid-level MR image at stress. Bullseye plots of MBF (ml/g/min) (c) at rest and (d) at stress, and (e) MPRI calculated from the NTH_*f*_ method.

**Fig 4:**
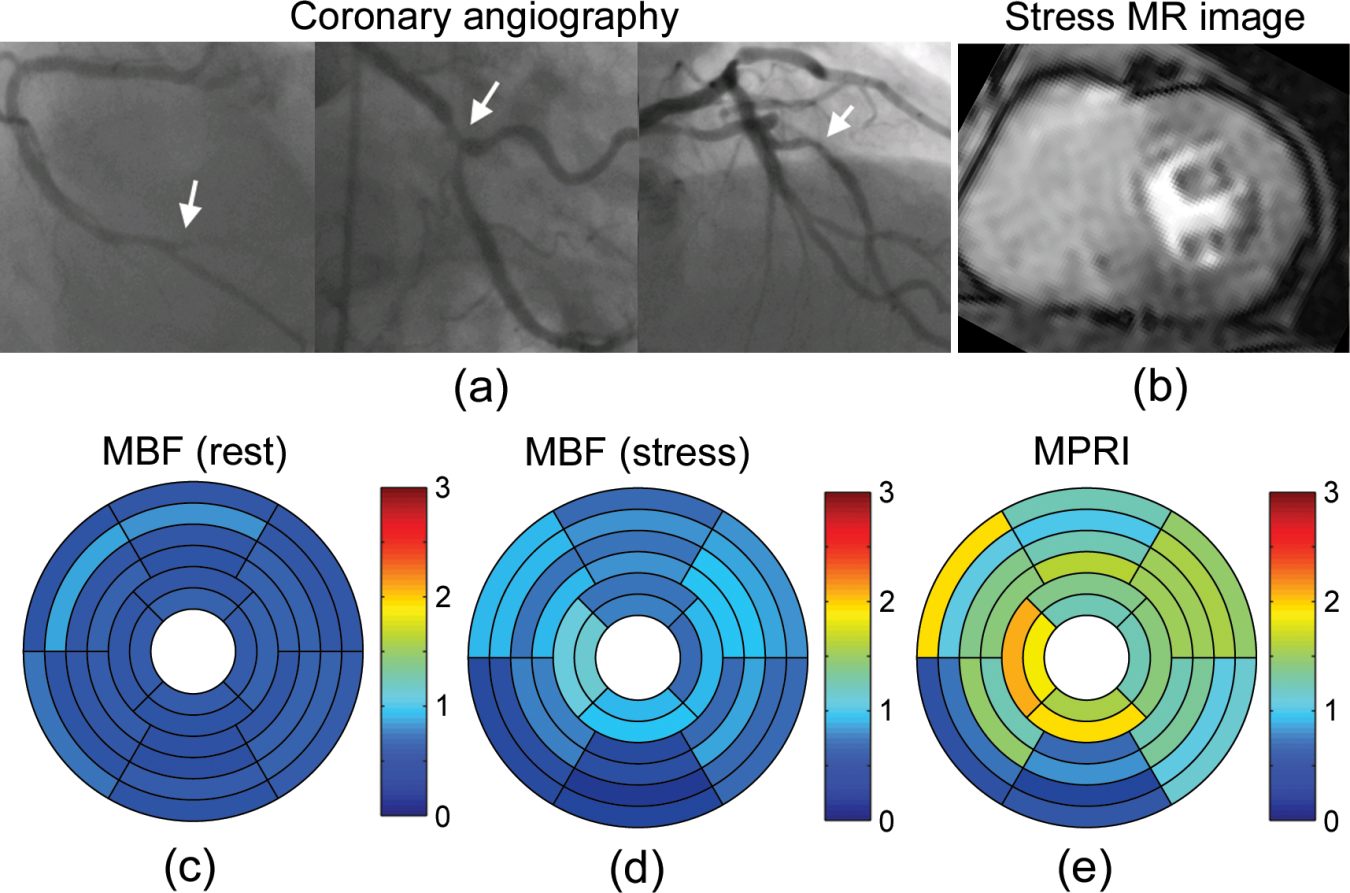
(a) Selective coronary angiography in a representative patient (66-year-old male) with severe triple-vessel disease (arrows). (b) First-pass enhancement mid-level MR image at stress showing a qualitatively delayed and decreased delivery of contrast agent to the inferior segments and milder delayed delivery to the anterior segments. Bullseye plots of MBF (ml/g/min) (c) at rest and (d) at stress, and (e) MPRI calculated from the NTH_*f*_ method.

**Fig 5:**
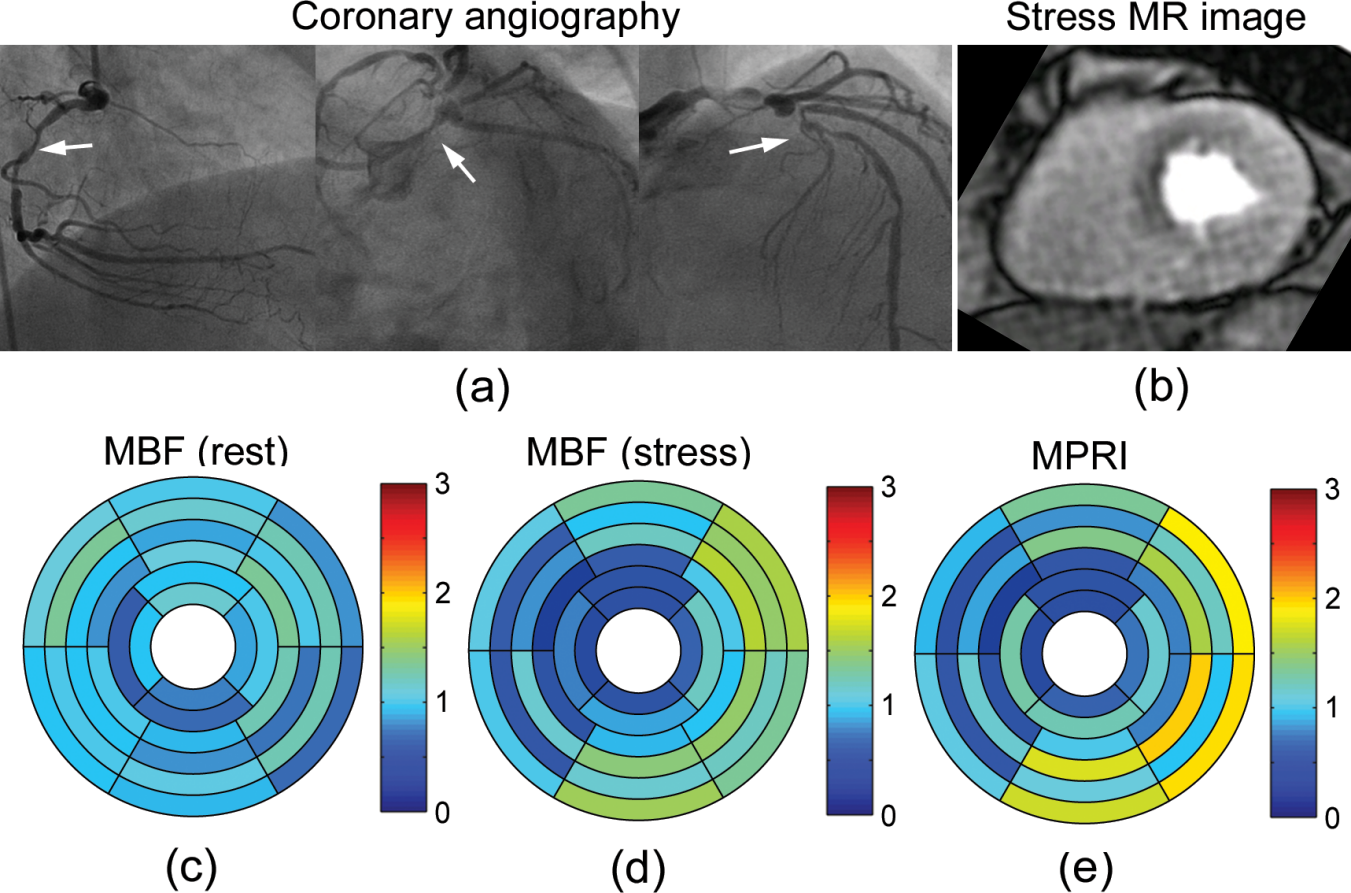
(a) Selective coronary angiography in a representative patient (65-year-old male) with severe triple-vessel disease (arrows). (b) First-pass perfusion mid-level MR image at stress showing relatively decreased flow to the anterior and anteroseptal wall. Bullseye plots of MBF (ml/g/min) (c) at rest and (d) at stress, and (e) MPRI calculated from the NTH_*f*_ method, showing more extensive perfusion abnormality.

The perfusion results for a representative patient (83-year-old female) with angiographically normal coronary arteries (Fig 3A) are shown in Fig 3. First-pass CMR perfusion imaging demonstrated a qualitatively uniform delivery of contrast agent to the heart wall (Fig 3B). The mean MBFs at rest were 0.98 ± 0.24 ml/g/min in LAD territory, 1.02 ± 0.15 ml/g/min in RCA territory, and 0.91 ± 0.16 ml/g/min in LCX territory (Fig 3C). The mean MBFs at stress were 1.63 ± 0.39 ml/g/min in LAD territory, 1.67 ± 0.37 ml/g/min in RCA territory, and 1.27 ± 0.31 ml/g/min in LCX territory (Fig 3D). The mean MPRIs were 1.69 ± 0.26 in LAD territory, 1.64 ± 0.22 in RCA territory, and 1.40 ± 0.26 in LCX territory (Fig 3E). On the other hand, Fig 4 shows the results for a representative patient (66-year-old male) with a history of hypertension, hyperlipidemia, diabetes mellitus and known coronary artery disease with prior stents, on maximal medical therapy. Coronary angiography demonstrated severe triple-vessel disease (Fig 4A). First-pass CMR perfusion imaging demonstrated a qualitatively delayed and decreased delivery of contrast agent to the inferior segments, consistent with ischemia or scar. There was also a suggestion of qualitatively milder delayed delivery to the anterior segments at stress (Fig 4B). The mean MBFs at rest were 0.60 ± 0.12 ml/g/min in LAD territory, 0.54 ± 0.08 ml/g/min in RCA territory, and 0.60 ± 0.07 ml/g/min in LCX territory (Fig 4C). The mean MBFs at stress were 0.85 ± 0.15 ml/g/min in LAD territory, 0.52 ± 0.30 ml/g/min in RCA territory, and 0.81 ± 0.18 ml/g/min in LCX territory (Fig 4D). The MPRIs were 1.45 ± 0.35 in LAD territory, 0.97 ± 0.55 in RCA territory, and 1.36 ± 0.18 in LCX territory (Fig 4E). Fig 5 shows another representative patient (65-year-old male) with angiographically severe triple-vessel disease (Fig 5A). First-pass CMR perfusion imaging showed relatively decreased flow to the anterior and anteroseptal wall (Fig 5B). The mean MBFs at rest were 1.00 ± 0.20 ml/g/min in LAD territory, 0.91 ± 0.11 ml/g/min in RCA territory, and 1.02 ± 0.24 ml/g/min in LCX territory (Fig 5C). The mean MBFs at stress were 0.73 ± 0.36 ml/g/min in LAD territory, 0.95 ± 0.38 ml/g/min in RCA territory, and 1.25 ± 0.29 ml/g/min in LCX territory (Fig 5D). The MPRIs were 0.75 ± 0.41 in LAD territory, 1.06 ± 0.44 in RCA territory, and 1.30 ± 0.52 in LCX territory (Fig 5E).

### Comparison of NTH_*f*_ and AATH Perfusion Estimates

**Fig 6:**
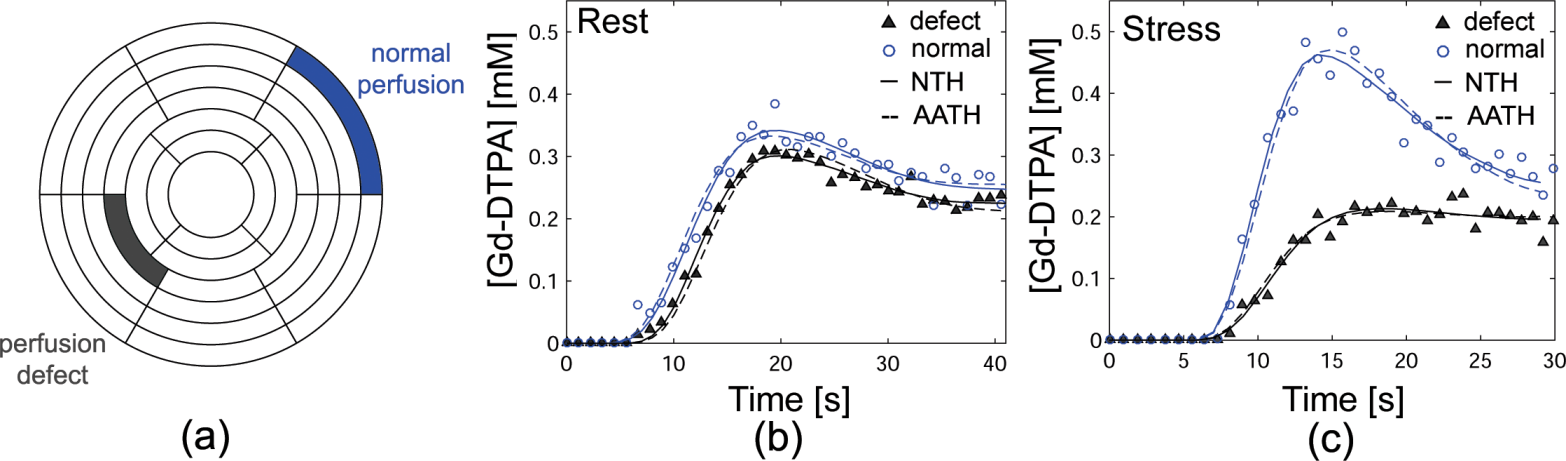
(a) Bullseye plot indicating representative segments of normal perfusion (blue) and perfusion defect (gray) in the patient with severe triple-vessel disease in Fig 5. Gd-DTPA concentration ([Gd-DTPA]) first-pass perfusion time-curves at (b) rest and (c) stress in the normal (blue circle) and defect (gray triangle) myocardium segments shown in (a) with best fit estimated plots from the NTH_*f*_ (solid line) and AATH (dashed line) models. Estimated MBFs (ml/g/min) using the NTH_*f*_ model at rest (stress) were 0.81 (1.56) in a normal segment and 0.99 (0.49) in a perfusion defect segment, and estimated MBFs using the AATH model at rest (stress) were 0.97 (1.30) in a normal segment and 0.93 (0.57) in a perfusion defect segment.

Fig 6 shows representative Gd-DTPA concentration ([Gd-DTPA]) first-pass perfusion time-curves in representative normal and perfusion-defect segments (Fig 6A) of the patient shown in Fig 5. The estimated MBFs at rest using the NTH_*f*_ and AATH models were 0.81 and 0.97 in the normal segment, and 0.99 and 0.93 in the perfusion-defect segment, respectively (Fig 6B). The estimated MBFs at stress using the NTH_*f*_ and AATH were 1.56 and 1.30 in the normal segment, and 0.49 and 0.57 in the perfusion defect segment, respectively (Fig 6C). Note that the solid and dashed lines in Fig 6B and 6C indicate the predicted plots from the best-fit NTH_*f*_ and AATH models, respectively.

**Fig 7:**
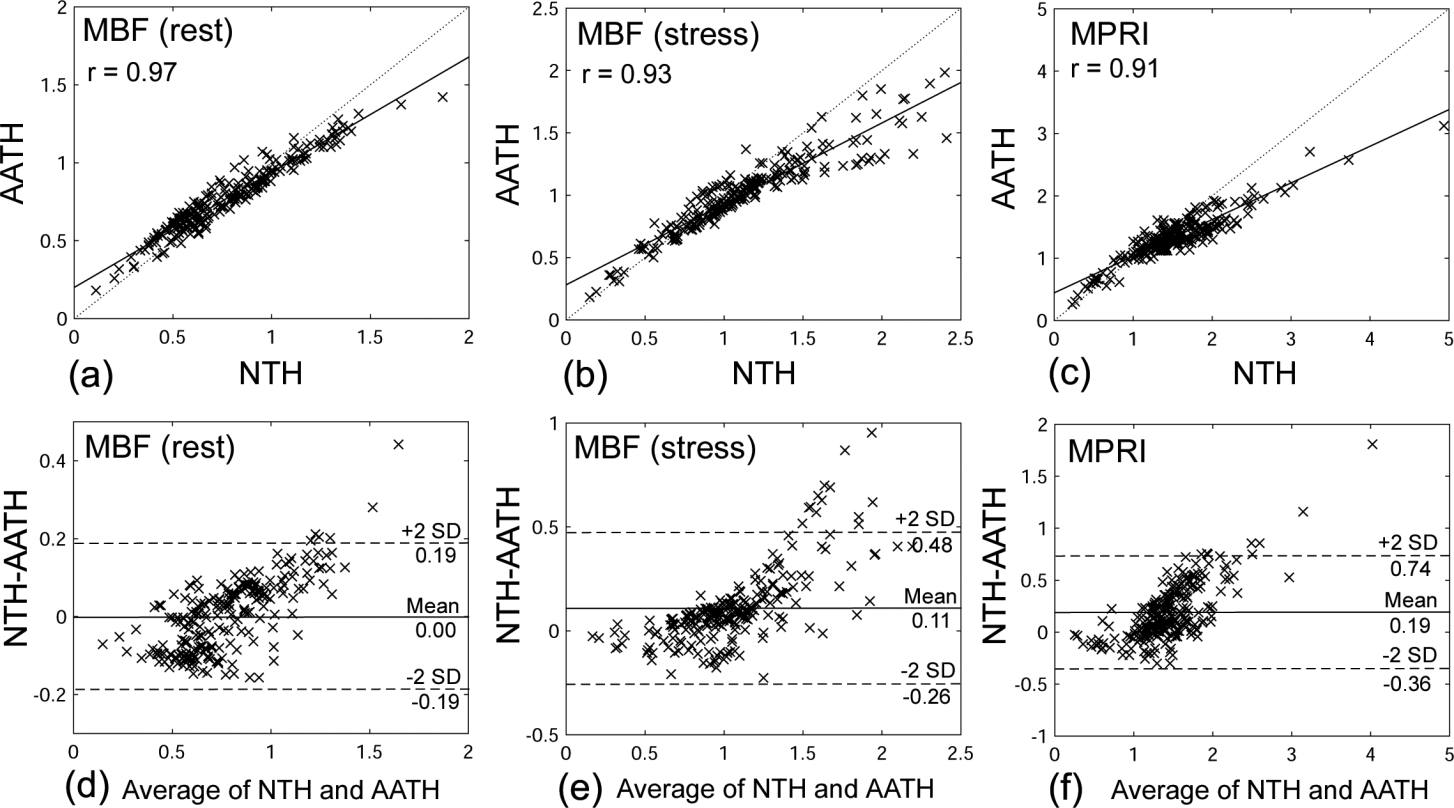
The correlation plots of MBFs from the NTH_*f*_ and AATH at (a) rest and (b) stress. (c) The correlation plot of MPRIs from the NTH_*f*_ and AATH. p < 0.001 for all. Bland-Altman plots of MBFs from the NTH_*f*_ and AATH at (a) rest and (b) stress. (c) Bland-Altman plot of MPRIs from the NTH_*f*_ and AATH.

We found strong correlation between the results of the NTH_*f*_ and AATH models. The correlation coefficients of MBFs calculated with the NTH_*f*_ and AATH models were r = 0.97 at rest (Fig 7A), r = 0.93 at stress (Fig 7B), and r = 0.91 (MPRI; Fig 7C). p < 0.001 for all. In particular, the MBFs estimated from the NTH_*f*_ model were higher than those from the AATH model at the higher flows (e.g., MBF > 1). The results of representative Bland-Altman plots are shown in Fig 7D–7F. The mean RSS values for 32 myocardial segments were smaller in the NTH_*f*_ (0.0068 ± 0.008 at rest and 0.0071 ± 0.0065 at stress) than the AATH (0.0119 ± 0.0136 at rest and 0.0116 ± 0.0079 at stress), showing a high goodness of fit in the NTH_*f*_.

### Noise Stability Estimation

**Fig 8:**
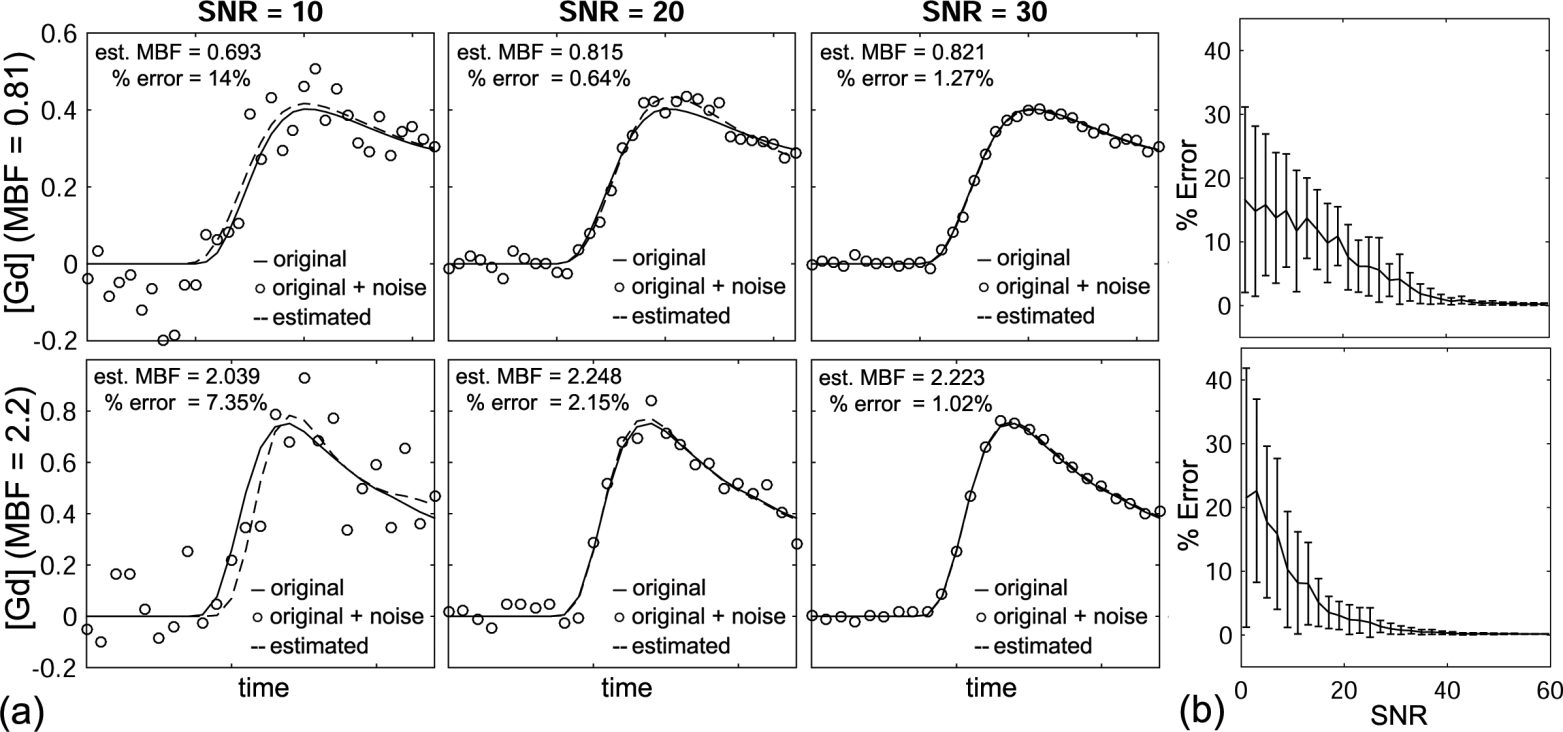
Plots simulated with (top) MBF = 0.81 and (bottom) MBF = 2.2. (a) Simulated (or original; solid line), White Gaussian noise added (circle), and estimated from NTH_*f*_ (dashed line) plots are shown at SNR = 10, 20, 30. (b) Percent errors for a range of SNR = 0–60 showing average and two standard deviation error bars (each SNR was repeated 30 times).

Fig 8A shows representative simulated plots at SNR = 10, 20 and 30, and corresponding fitting plots from the NTH_*f*_ model. Over all, the percent errors at MBF = 0.81 and 2.20 are up to 16.6 ± 14.5% and 21.5 ± 20.3% (mean ± 2 SD) for a range of SNR = 0–60, respectively (Fig 8B).

## Discussion

This study has presented a new approach for numerical implementation of the TH model for analysis of first-pass tracer passage dynamics from MRI studies. While the AATH model considers the tracer exchange along the capillaries as a separate unit, with a fixed discretization of both the space and time dimensions, we have considered the exchange from the frame of reference of blood flowing as flexible packets moving along the capillaries. We evaluated this model on a small set of patients with suspected CAD and found statistically significant differences of MPRI between the myocardial segments supplied by coronary arteries with different severities of stenosis, as assessed with coronary angiography (Table 2). We found strong correlations (r > 0.9) of the perfusion results from these two models. However, we also found that the MBFs estimated from the NTH_*f*_ model were higher than those from the AATH model at the higher flows (MBF > 1), as clearly seen in Fig 7A–7C. We believe that this difference in computed perfusion between the two methods at higher flows is because the NTH_*f*_ model approach allows more stable calculations of the transit of tracer through myocardial tissue, particularly at higher curvature regions such as the beginning of the ascending part and the peak of the concentration-time curves which play important roles in the estimation of myocardial perfusion. The results of the RSS confirmed a higher goodness of fit of the NTH_*f*_ model compared to the AATH model through all time points.

The results summarized in Table 2 for the patients with CAD show that the mean MBFs in segments with insignificant arterial stenosis are 0.80 ± 0.33 ml/g/min at rest and 1.25 ± 0.45 ml/g/min at stress, respectively, and the corresponding mean MPRI is 1.68 ± 0.54. These values are lower than other published results [22]; this might be due, in part, to differences in cardiac workload for the individuals (MBF correlates linearly with the rate-pressure product [23], ranging from 5160 to 12225 in this study), differences between regadenoson- and exercise-induced changes in perfusion, or potential bias resulting from residual contrast agent from the previous (rest) scan, which was separated by 10 minutes. Additional studies with more subjects will be needed to explore this further. The use of an estimated “small vessel” hematocrit for calculation of the plasma space in the microcirculation will also result in a different calculated flow than the use of the large vessel hematocrit value; our use of an estimated factor of 0.7 for the difference of large and small vessel hematocrits would result in a corresponding difference in the calculated tissue blood flow, as compared with the results of other studies that have not included this factor.

In this study, we have assumed that the water is moving rapidly enough between compartments to be in the "fast exchange" limit, so that the observed changes in the tissue relaxation rate can be used to estimate the total concentration of contrast agent in the tissue. The question of whether or not the water exchange is sufficiently rapid for this assumption to hold is a well-known (and not yet fully resolved) issue in contrast-enhanced MRI, and it can cause a systematic error in the calculation of perfusion or the related parameters if it does not hold. However, the effects of water exchange on observed relaxation can be modeled and appropriate corrections applied to the estimated concentration values, if we are not in the fast exchange regime for a given application situation. For a given set of derived concentration-time data, the corresponding perfusion analysis would then proceed as above.

As is commonly done, we have modeled the microcirculation as if it were characterized as just composed of capillaries, with a single effective length. This approach could be readily generalized by modifying the model, although at a cost of increased computation time, and with a potential for increased numerical instability: 1) For an assumed distribution function of values of MTT, rather than a single effective "mean" value, we could compute the net tissue concentration as a weighted average of the tissue concentrations expected for a corresponding set of different values of MTT. While this would be expected to result in a corresponding broadening of the predicted time course of the tracer concentration-time curves, the overall behavior would still be similar to that computed for a single MTT at the mean value. Thus, this is not likely to result in a sufficiently better fit to the data to justify the additional associated parameter fitting. 2) If a portion of the volume of the microcirculation is considered to be allocated to arterioles and venules, which have some blood volume but do not participate in tracer exchange with the extravascular space, the effective value of PS will be reduced compared to that for a model of the microcirculation with just capillaries, with the result that the value for the capillary PS calculated from the model fit will be expected to be greater. However, the overall associated resulting improvement in the fit to the data is not likely to be good enough to justify the additional associated parameter fitting.

The potential clinical utility of this method for determining absolute, rather than qualitative, perfusion is clearly demonstrated in the setting of multi-vessel disease. In particular, in the presence of triple-vessel disease, with "balanced lesions", we may tend to underestimate the extent or severity of perfusion reserve in the affected regions with simple qualitative or semiquantitative approaches to perfusion assessment, as was the case in the patients illustrated in Figs 4 and 5. In particular, the quantitative analysis made possible the demonstration of a stress-induced decrease in perfusion of the more severely affected regions, likely representing the presence of a “steal” phenomenon. Having an efficient and robust means for carrying out the associated calculations should help to make such quantitative CMR perfusion assessments more clinically practical.

The inclusion in our modeling approach of the effects of the transit of the tracer from the AIF observation point to the tissue of interest is not an intrinsic part of the TH model per se, but it allows for a more realistic modeling of the tissue response than simply assuming an effectively instantaneous transit of the tracer from the AIF observation point. Using a coupled estimate of the effects of delayed transit and associated broadening of the tracer bolus allows incorporation of both effects in the modeling with only one associated parameter value.

In comparison with simpler models, analysis of first-pass enhancement kinetics with the TH model can be a little more complicated. However, the removal of the need to effectively assume an instantaneous transit of some tracer through the tissue allows the TH to provide a more realistic model of the transit than simple compartment models, particularly for higher temporal resolution observations. The use of an analysis based on underlying physiological variables, rather than phenomenological models, allows for derivation of parameter values that are more independent of the observation conditions, e.g., the apparent dependence of the “extraction fraction” of the tracer on the rate of blood flow can be avoided with the corresponding calculation of PS and volume fractions. The use of an iterative fitting of the underlying model parameters is more robust to noise in the data than the use of more direct deconvolution approaches to finding an impulse response to an idealized tracer bolus, which tend to be more vulnerable to noise.

In the application of these first-pass enhancement imaging perfusion methods to the analysis of myocardial perfusion, the effects of any respiratory motion during the bolus transit will need to be compensated for, either manually or more automatically, as with any such first-pass perfusion imaging. In fitting the blood flow parameter, it is the earlier data, during the inflow of blood to the tissue, that is most important; however, the later data points will be more important for estimating the values of PS and the extravascular space. In particular, it will be important to avoid any contamination of the subendocardial segments by signal from the strongly enhancing intracavitary blood pool. It is useful, however, to separately analyze the subendocardial and subepicardial portions of each wall segment (resolution permitting), as there is typically a significant transmural difference in the regional perfusion in ischemia, as was seen in our limited patient sample.

There are several limitations of this study. First, we evaluated our CMR perfusion modeling approach on a sample of only 8 patients with suspected coronary artery disease, of whom only 2 were women, and the results were compared with the corresponding conventional angiographic severity of coronary artery stenosis in each corresponding coronary artery territory, assigned to individual segments as conventionally recommended.[21] This was a prospective study, in which all subjects underwent MR imaging, followed by a clinical diagnostic invasive coronary angiography examination on the same day, in order to provide more accurate results of the severity of underlying coronary artery stenosis from the associated angiography examination. Unfortunately, we had logistical difficulties in coordinating the different studies (due to limited MR imaging/clinical coronary angiography examination schedules and limited patient availability), which limited recruiting not only of gender-balanced pairs of patients but also the total number of patient subjects included in the study. Functional assessment of the physiologic significance of observed stenoses by FFR was only available for a minority of the observed lesions. Although the number of patients in this study was small, and the results were compared by pooling the results for the relatively large areas corresponding to the three coronary artery territories (which might have some variability in the specific coronary artery blood supply to individual myocardial segments), the encouraging results suggest a clinical potential of this method for identifying physiologically significant coronary artery disease. Further larger studies in a larger population are warranted to validate this approach. Second, the results were also compared with those from the AATH model. Although we found strong correlations between the results from the NTH_*f*_ and AATH models (Fig 7A–7C), the MBFs estimated from the NTH model were overall higher than those from the AATH model, particularly at higher perfusion, where the AATH model may have problems. However, there are difficulties in using these results for a quantitative validation, since an underlying ground truth is not well defined. Third, rest and stress scans were not performed in random order, which might ideally have been done, in order to minimize any potential bias resulting from residual contrast agent from the previous scan (which was separated by 10 minutes); this was done to avoid potential confounding effects from any residual vasodilating effects of the regadenoson [24]. This may have led to bias in our estimates. Fourth, the capillary hematocrit, Hct_c,_ is not readily directly measured, while the large vessel hematocrit, Hct, can be measured by simply sampling blood from a large vessel. Thus, we generally rely on estimates for the relative difference derived from the literature [16]; here, we assumed that *Hct*_*c*_ = 0.7 × *Hct*, where Hct was individually measured in this study.

In conclusion, taking a new approach to perfusion analysis that combines aspects of the TH model and a Lagrangian approach to modeling transcapillary exchange with flexible distance steps along the capillary allows efficient quantification of the transit of tracer through tissue while still retaining a relatively simple model of the exchange during the transit. There is a potential to apply this new approach to the modeling of tracer exchange to identify physiologically significant coronary artery disease, using calibrated CMR imaging methods to observe the first-pass enhancement of the myocardium by a bolus of contrast agent, as demonstrated in this study. Future work would include further validation of these results in a larger patient population.

### Compliance with Ethical Standards

This study was approved by our Institutional Review Board at the NYU School of Medicine, and all subjects provided written informed consent before the procedure.

#### Ethical approval (in case humans were involved)

All procedures performed in studies involving human participants were in accordance with the ethical standards of the institutional and/or national research committee and with the 1964 Helsinki declaration and its later amendments or comparable ethical standard.

#### Informed consent

Informed consent was obtained from all individual participants included in the study.

## Supporting Information

S1 File Subject’s MBF and MPRI from NTH_*f*_ and AATH methods. Average MBF and MPRI values were calculated in each region of LAD, RCA, and LCX, using NTH_*f*_ and AATH methods. Stenosis [%] indicates the severity of coronary artery stenosis determined from the invasive coronary angiography examination by a cardiologist after the procedure and expressed as a percent reduction in vessel diameter.
(XLSX)
